# Ngram-Derived Pattern Recognition for the Detection and Prediction of Epileptic Seizures

**DOI:** 10.1371/journal.pone.0096235

**Published:** 2014-06-02

**Authors:** Amir Eftekhar, Walid Juffali, Jamil El-Imad, Timothy G. Constandinou, Christofer Toumazou

**Affiliations:** Centre for Bio-Inspired Technology, Part of the Department of Electrical and Electronic Engineering, Imperial College London, South Kensington Campus, London, United Kingdom; University of Ulm, Germany

## Abstract

This work presents a new method that combines symbol dynamics methodologies with an Ngram algorithm for the detection and prediction of epileptic seizures. The presented approach specifically applies Ngram-based pattern recognition, after data pre-processing, with similarity metrics, including the Hamming distance and Needlman-Wunsch algorithm, for identifying unique patterns within epochs of time. Pattern counts within each epoch are used as measures to determine seizure detection and prediction markers. Using 623 hours of intracranial electrocorticogram recordings from 21 patients containing a total of 87 seizures, the sensitivity and false prediction/detection rates of this method are quantified. Results are quantified using individual seizures within each case for training of thresholds and prediction time windows. The statistical significance of the predictive power is further investigated. We show that the method presented herein, has significant predictive power in up to 100% of temporal lobe cases, with sensitivities of up to 70–100% and low false predictions (dependant on training procedure). The cases of highest false predictions are found in the frontal origin with 0.31–0.61 false predictions per hour and with significance in 18 out of 21 cases. On average, a prediction sensitivity of 93.81% and false prediction rate of approximately 0.06 false predictions per hour are achieved in the best case scenario. This compares to previous work utilising the same data set that has shown sensitivities of up to 40–50% for a false prediction rate of less than 0.15/hour.

## Introduction

Epilepsy is a neurological disorder that affects approximately 1% of the world’s population. It is characterised by seizures, which can manifest in several ways, from simple loss of awareness to more severe motor movements with loss of consciousness. A multitude of fields have studied the underlying mechanisms behind seizures, looking at the brain from multiple perspectives including bottom-up (i.e. local neuronal microcircuits) to global approaches (i.e. network activity monitored through local field potentials or EEG).

The goal of this work is in the detection and prediction of the epileptic seizure. Prediction has seen work ever since the 1950s applying linear, nonlinear (state-space) or multivariate analysis techniques to EEG and derivatives of it [1]–[3]. The ability to predict a seizure would allow for intervention strategies [4]–[6] to be administered for those patients where medication or surgery has had little or no effect. At present, 70% of people with epilepsy can have it controlled with the correct anti-epileptic drugs. For those not helped by medication there are options, including surgery, which is successful in up to 70% of cases (http://www.epilepsynse.org.uk/).

Early work on seizure detection has predominantly focused on neonatal EEG. Seizures in neonates can be indications of Neonatal Encephalopathy (NE) - the manifestation of abnormal neonatal brain function - and can affect from 0.5–4 neonates per 1000 [7], [8]. However powerful, current methods [9]–[11] fail to aid in the detection of small seizures (several seconds) and can miss up to 50% of seizures if unsupervised (without expert monitoring) [12]. A recent study [13] analysed a significant number of time, energy and frequency domain features for the detection of seizures in adult data, comparing complexity and accuracy. Although significant, the results still show relatively low sensitivity and specificity to previous studies, with no indications of inter-patient variability.

More recent detection work, looking at wave morphology changes [14] have shown an improved sensitivity of 81% and false detection rate (FDR) of 0.18/h on intracranial EEG. The same work also presented a comprehensive comparison with some previous results such as the Aarabi system, who originally used linear correlation features in neonatal EEG [15] but in the most recent study fuzzy rules, showing a sensitivity of 98.7% and FDR of 0.27/hand. Previous work typically shows sensitivities greater than 79% and FDRs of approximately 0.08 to 0.14/h [14].

Similar to detection, there has been a wealth of work aiming to predict seizures [1], and more recently an influx of methods showing high sensitivities with statistical validation applied. Statistical validation being a more recent requirement of prediction methods motivated by the prediction review of Mormann [1]. This has established the generally accepted statistical framework [16], [17] that compares the results to a random predictor.

One of the first to separate temporal and frontal, and clinical and subclinical seizures showed sensitivities of 40–50% for a FPR of 0.15/h [17]. Some more recent to note include Aarabi et al who used a rule based system utilising nonlinear measures on intracranial EEG, and subsequently showed sensitivities of 80% and 90% for a prediction time of 30/50 mins, with FPR of 0.17/0.11/h respectively [18]. Other studies, have shown sensitivities of 80–88% with some level of significance and FPRs of of approx. 0.15/hour [19]–[21]. A recent study by Williamson et al was able to show only 15 false predictions in a 440 hour period (less than 0.04 FP/h).

At present, there are still avenues to explore for both prediction and detection of seizures. Prediction specifically has progressed significantly since [1] but there are still many unknowns. This has not prevented the immense amount of research in the area, applying many variations of algorithms, including some of the first-in-man studies [22]. However, there is a clear emphasis on the requirement for statistical validation of results versus a random predictor. In this paper we explore the application of a new method for both detection and prediction. This is based on a modified pattern recognition method adopting the N-gram algorithm that we have previously described in [23]. Developing this method into a real-time analysis environment, we explore (and quantify) its ability to detect and predict seizures. We also compare this against some of the most recently published results in the area.

## Symbolic Analysis of EEG Signals

Pattern recognition algorithms typically involve three stages: (1) data acquisition, (2) data representation and (3) decision making [24]. Data acquisition employs an analog front-end, including amplification, filtering and data conversion (analog to digital). Once acquired features can be extracted from the signal (either in the digital or analog domain), for example [25]. Representing data as features reduces the amount of data/information required and subsequently reduces the complexity of any further analyses. Typically this is then followed by a classification method, such as clustering. An excellent review of all these methods and the future perspective on this topic refer to [24] and references therein.

### N-grams

Traditionally applied to language models, an N-gram model extracts and counts the subsequences of a particular symbolic sequence [26], such as words in a body of text, i.e. a word search. These subsequences or patterns (i.e. phrases/sentences) can be predicted based on the probabilities of words occurring given the previous *n-1* words [27] (i.e. the Markovian nature of the sequence).

**Figure 1:**
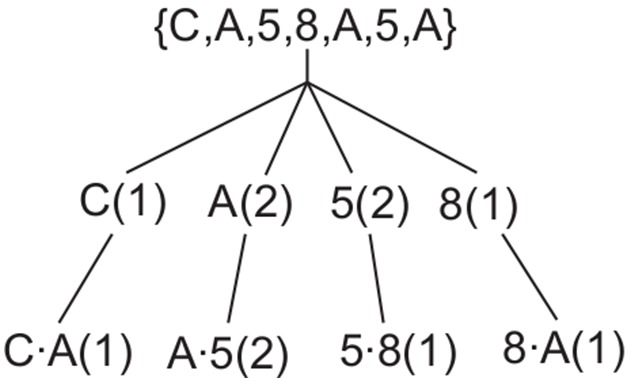
N-gram pattern sequence example for the sequence C, A, 5, 8, A, 5. A unigram and bigram are shown for this sequence, 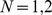 and their associated counts.

In an N-gram model -sequences of symbols are found within the data, setting up an N-gram tree with 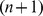 nodes each with a list of the combination of symbols and their count within the data. With this we can extract various measures to estimate signal complexity, transition probabilities and predict future sequences of symbols [28], [29]. So an example sequence 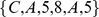 will have a bigram (N-gram of order 2) as per Fig. 1 where the symbol  is used as a symbol separator.

For pattern recognition, separation of this symbol sequence into meaningful patterns is the first step after symbolisation, i.e. it allows the formation of a pattern search tree. This is typically done using entropy and information theory methods [29] based on the probability of symbols following each other (eq. 1). The probability then of 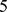 given 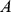, i.e. 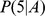, is 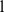 as all cases of symbol 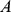 are followed by a 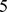. This process requires the N-gram tree to first be built, so can be computationally and memory demanding process depending upon the data size.
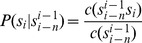
(1)


### Applications to EEG

Symbolic data analysis itself has been applied to time series in many applications including EEG analysis [30]–[32]. The process involves defining a mapping that translates a given data sequence 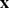 (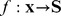) to a symbolic space 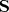 with 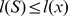, where 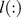 is the length of the enclosed vector. This mapping can be achieved in many different ways including representing 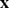 in a multidimensional state-space [28], [33]–[35]. Once symbolized the sequence is either clustered as patterns (such as words from character sequences) or a measure is applied to quantify symbolic dynamics, the latter typically employed using entropy-based measures [30], [36].

A few studies have looked at signal symbolic analyses as a way of quantifying seizure-related activity. The first, is a series of work by Hively et al. [34], [35], [37] that uses quantized time-series. These series are translated to a 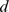-dimensional space using time-embedding [37], [38] which is then partitioned into bins. Over pre-defined time-windows the occurrence of the signal in these bins is counted and compared between a *base* or normal case and a *test* case using the 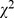 statistic and 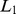 distance. These are compared to traditional measures of correlation dimension, mutual information and Kolmogorov entropy on model and real EEG data. They showed that the phase space symbol dynamics offered superior separation between pre-seizure and seizure state compared to the other measures.

Schindler et al [31] used the approach of ordinal time series analysis by windowing EEG data and observing the uniform set of sub-sequences that occur in that window. They observed cases of pattern changes prior to seizure but since the set of results presented were limited (in addition to time being normalized for all cases) it is difficult to truly assess the significance of the results. Finally, Eftaxias [36] used a binary representation; a threshold being a function of the mean, with the signal, when greater than, resulting in a 1, and less than, 0. This was followed by several entropy and information quantifiers including the Tsallis entropy and Symbol Fisher Information Measure (SFIM). Using these and other measures they are able to show evolutions in evoked rat seizures several minutes prior to onset and in human data discrimination between pre-seizure and seizure states.

The results of these studies have shown significant progress in symbolic analysis’ applicability to EEG-based time series, but have yet to quantify statistical prediction properties, including significance.

## Methods

**Figure 2:**
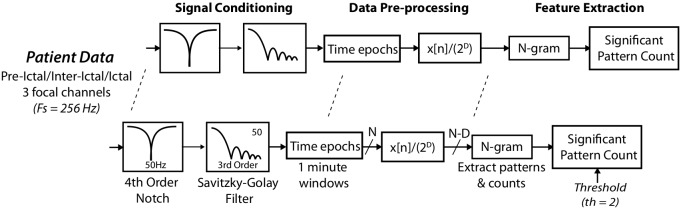
Algorithm system flow where the input data files for each patient are filtered, pre-processed then analysed with our N-gram method with anomalous activity detected using a pre-defined threshold on the pattern counts.

The aforementioned analyses methods are typically based on symbolic time series literature, while, as we previously mentioned, in language modelling this type of analyses uses an N-gram approach. In this section we describe our methodology (illustrated in Fig. 2).

### Signal Conditioning: *Filtering*

Our data contains 50 Hz mains noise so we apply a 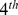 order Butterworth notch filter to each data set prior to analysis. In addition, a number of the data sets contain artefacts including electrode/amplifier saturation. A phase quadratic filter [35] was chosen, analogous to a Savitzky-Golay (SVG) smoothing filter, to remove these artefacts. This method applies a 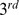 order smoothing function spanning over 101 sample points (50 before and 50 after a the signal point 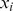). The SVG smoothing involves constructing a polynomial around these points with a least square fit and and using the central point, 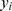 as the new smoothed version of 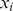. This is iteratively computed for each data point. The smoothed signal is removed from the original data set to leave us with a relatively artefact-free signal.

### Data Pre-processing

**Figure 3:**
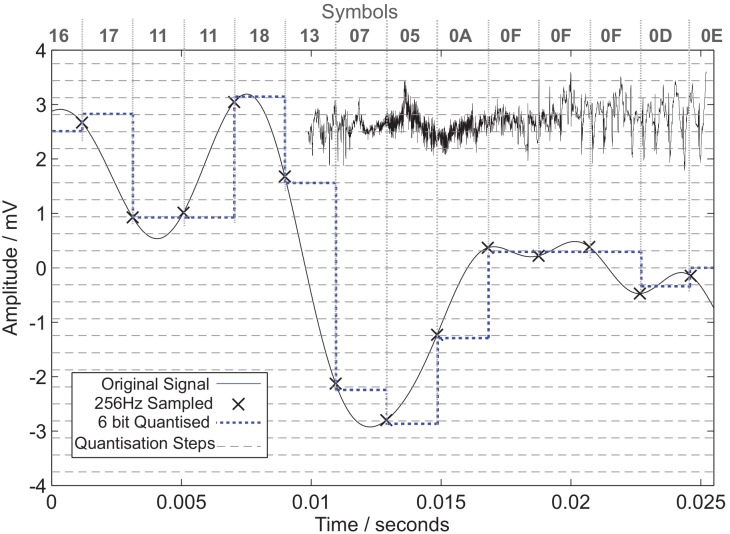
Traditional quantization (6 bit sampled at 256 Hz) on part of the sample EEG signal shown. Also shown are the equivalent hexadecimal symbol representation of this data.



Once filtered, the signals are separated into time windows and re-quantized. In data acquisition systems a signal is typically quantized into 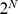 levels between two predefined reference points (2) thus reducing the signal amplitude resolution. This is equivalent to a symbolic representation in a one dimensional (amplitude) space (Fig. 3). Rather than define 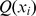 as an integer, we can represent it as a symbol 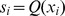, which for case of simplicity is defined as the hexadecimal representation of the quantized signal’s binary value.

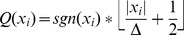
(2)

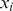
 being a data point, 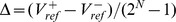, where 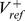 and 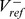 are the analog-to-digital converted reference positive and negative voltages, and 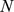 the bit level representation.

Although typical hardware quantization employs binary (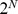) levels computationally finer amplitude resolution can be achieved by having a non-binary divisor. EEG data, depending upon the application, is already quantized at 8–16 bit which, when stored, is represented at its lowest level in binary, but in a higher level of abstraction can be represented in hexadecimal (HEX).

The re-quantized signal is generated by converting the already quantized signal, 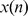, to a lower resolution according to 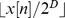. For example, conversion of a 16 bit number to an 8 bit requires 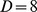, i.e. truncating the least significant bits (LSBs). The new binary sequence is then subjected to our modified N-gram methodology. Fig. 3 illustrates an example of our symbolic representation.

### Multiresolution N-gram

**Figure 4:**
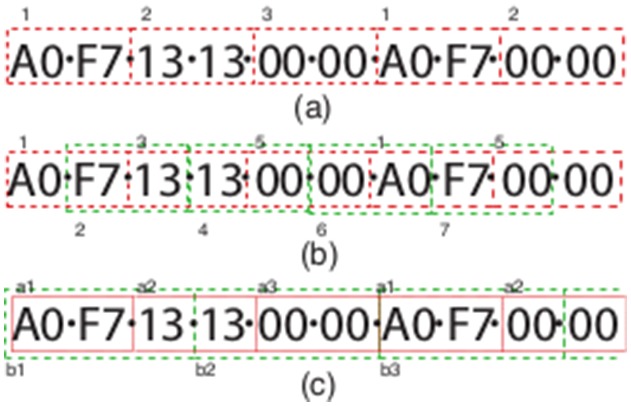
An example symbol sequence partitioned in three of the proposed methods: (a) using a single pattern length of 2 (b) using an overlapping pattern length of 2 and (3) multiple patterns non-overlapping (a = 2, b = 3). The index of each unique pattern is also labelled.

The aim of our method is to utilise the principles of an N-gram model but without the requirements of building an N-gram tree first. Instead we build the tree as we progress in time through a fixed window (1 minute), defining the patterns and associated counts. While building the tree we extract significant pattern counts. There are four ways we do this, three of which are illustrated in Fig. 4:

1. *Non-overlapping, Single Pattern:* This splits each window into a series of successive non-overlapping patterns of one pre-defined length (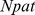). For the example shown in Fig. 4 and 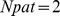, the generated patterns are: 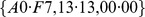 of which 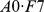 and 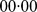 have a count of 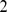.
2. *Overlapping, Single Pattern:* As in *(1)*, we have one pattern length, but now with each pattern overlapping. For the example shown and 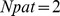 this would generate: 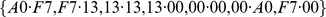 of which again 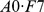 and 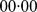 have a count of 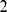.
3. *Non-overlapping, Multiple Patterns* Here we use multiple patterns where, as in method *(1)*, each pattern size does not overlap with each other, although other pattern sizes can, as in method *(2)* (see Fig. 4). For pattern sizes of 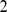 and 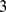 this would generate the patterns found in *(1)* and 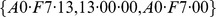.
4. *Overlapping, Multiple Patterns* This combines method *(3)* with the use of overlaps as defined in method *(2)* and results is several overlapping patterns shown in Table 1.

**Table 1:** Patterns and counts for method (d) using 2 pattern sizes (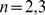).

Pattern (n = 3)	Count	Pattern (n = 2)	Count
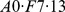	1	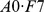	2
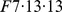	1	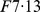	1
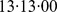	1	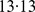	1
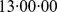	1	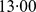	1
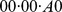	1	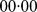	2
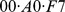	1	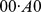	1
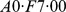	1	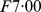	1
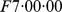	1		

The above four mentioned methods are the first step in the process of analysis. For the overlapping cases, as we want to establish the unique set of patterns in a symbol sequence we need to establish which overlapping patterns to count and which to discard (note the results in Table 1 does not show this).

**Figure 5:**
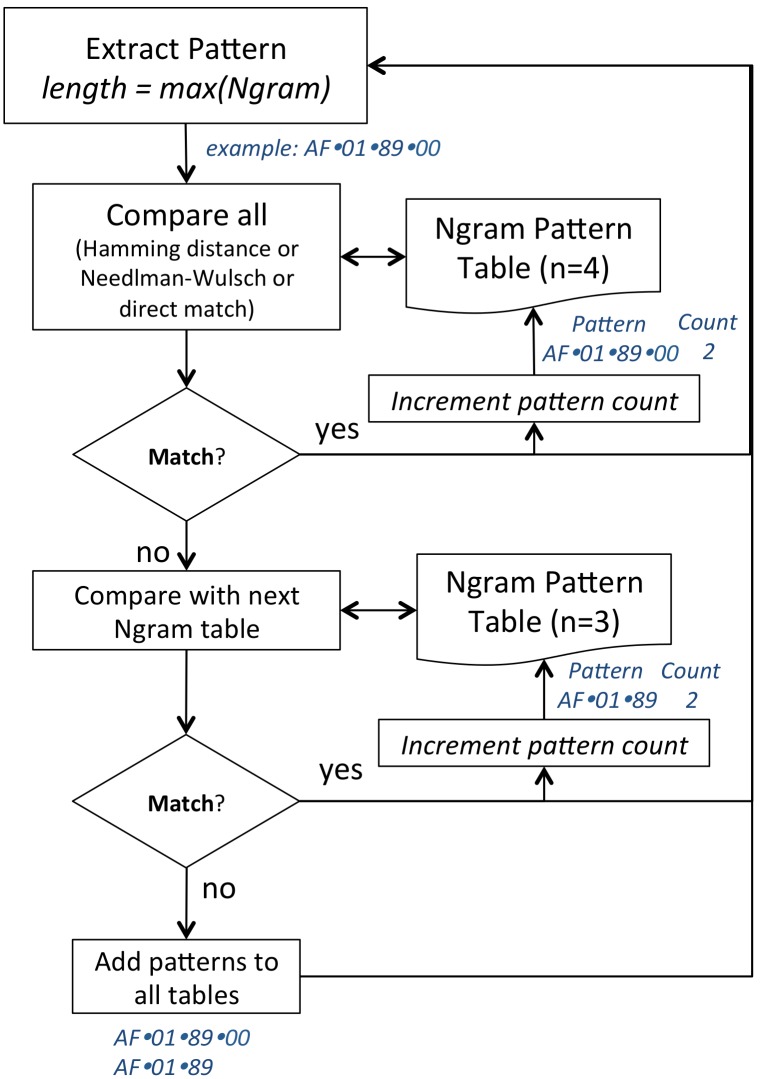
A simplified example of the process for generating unique patterns for Ngram sizes of 3 and 4. The pattern is initially compared against a table containing the largest patterns (i.e. length 4). If found, the pattern count is incremented, otherwise the pattern is compared to the next largest pattern (i.e. length 3). If no match is found here both are added to the tables.

Unique patterns are achieved during the process of extracting patterns in the time window. During this process we first look for the largest pattern and if it has occurred previously we skip the remaining smaller ones; the largest pattern that repeats is considered dominant over any others which overlap with it. Significant patterns are then defined as those that occurred more than 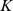 times. This is described in Fig. 5 for an example of Ngram sizes 3 and 4. To facilitate continuous-time analysis the data is segmented into windowed epochs, each quantized and then subjected to this pattern extraction methodology (Fig. 2).

**Figure 6:**
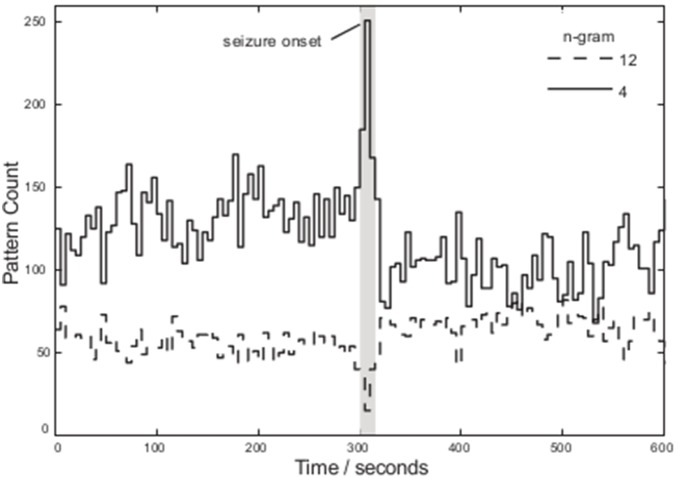
The result of applying the unique N-gram pattern search for pattern lengths 12 and 4 (method *(3)*) using a binary size of 4 bit over 5 second windows for a sample EEG signal.

As an example, during a seizure onset (annotated in Fig. 6), the number of unique large patterns reduces (due to periodic features), while the smaller pattern sizes increase somewhat. We found at this stage of the algorithm that the increase in smaller patterns is partially due to variations in patterns symbols that are in fact subsets of the larger pattern lengths; a member of the smaller pattern size varies in symbol classification and it is missed as being a subset of the larger pattern. This can be partially resolved using similarity metrics.

*Similarity* is determined by looking at correlation metrics between found patterns of the same length. Thus patterns can be clustered together if within a certain similarity threshold. In this work we consider two similarity metrics.

The first measure is the percentage of similar symbols of two sequences using the inverse Hamming distance (HD). For patterns of equal lengths 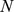, with symbols 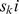 and 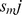, 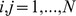, the inverse HD for these two patterns is 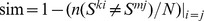 where 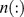 is the number of symbols that meet the argument enclosed.

The second similarity index we consider is based on the Needleman-Wunsch (NW) dynamic programming algorithm [39] used in bioinformatics (e.g. genetic and amino acid sorting applications). The advantage of this algorithm over the HD is that the two patterns need not be the same length. To use the NW algorithm we first remove all symbols not common to both patterns. We then apply the NW algorithm to determine a similarity score using a marking of 2 for equal symbols, 0 otherwise and a gap penalty of −1 (which occurs if patterns are different sizes). We then normalize the score by the total length of the two patterns.

For example, two sequences, 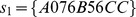 and 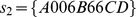 will result in a HD and NW index of 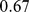. However, if 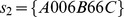 only the NW index generates a value of 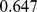. When constructing the unique pattern list in a data epoch we use one of these two similarity indices rather than an exact pattern match to cluster and count similar patterns. Values of similarity 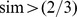 are clustered together.

**Figure 7:**
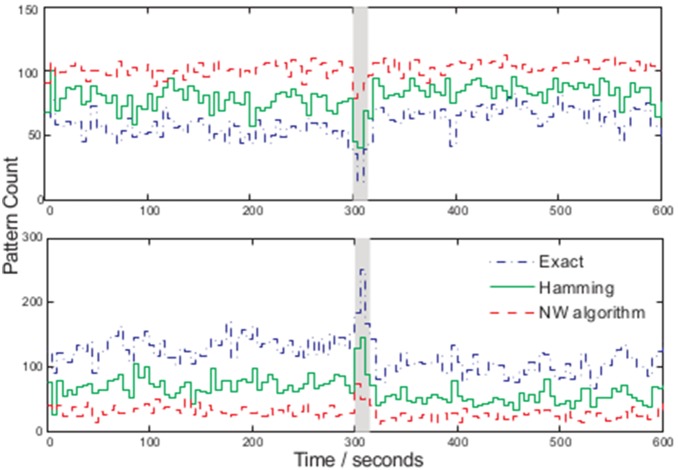
Comparison of methods for assessing sequence similarity in the multiresolution N-gram process, with pattern sizes of 12 (top) and 4 (bottom) over 5 second windows (method *(3)*).

Comparing the Hamming distance and NW algorithm to exact pattern matching we now observe an average increase in the number of larger patterns and decrease in the number of smaller ones (Fig. 7). Given the simpler implementation (and computation time) of the inverse Hamming distance we utilise this metric in our pattern sorting.

### Detection/Prediction Threshold

Once processed we have a count of the unique patterns over a pre-defined window (such as those illustrated in Figs. 6 and 7). The next step is to determine a threshold to use for prediction and detection. There are two types of threshold we use, static and dynamic. The static is a fixed multiple (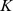) of the standard deviation (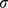) of the signal, 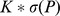, where 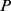 is the pattern count over the pre-defined window.

The dynamic threshold is a function of the moving average of the pattern count, 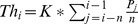, where 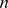 is the number of past values to use and 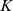, as in detection, is a constant that is trained in the optimisation and training process.

**Figure 8:**
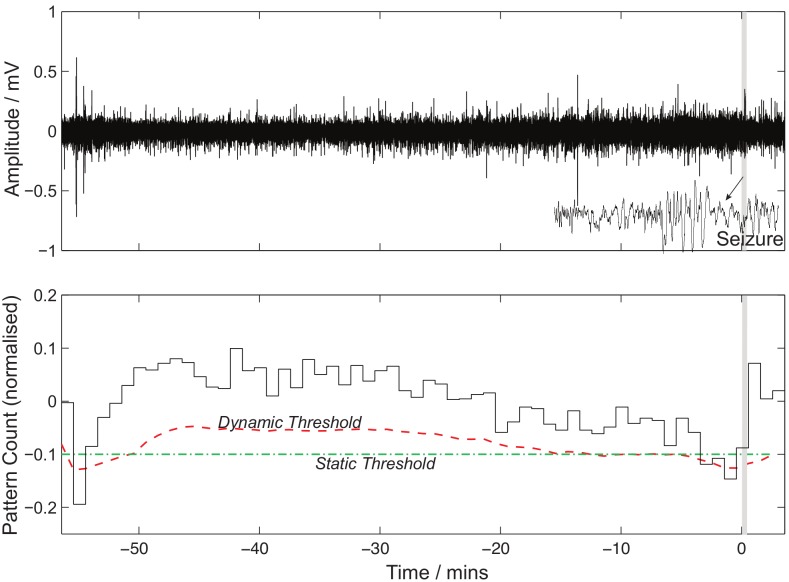
An example of Patient-15′s pattern change (bottom) and corresponding normalized EEG (top) with seizure occurring at time, 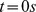. Also shown is a zoomed in view of the seizure on the EEG (top) and the optimal dynamic and static prediction thresholds.

To facilitate the application of the threshold on the ictal and interictal data we normalize the pattern count to the average of a randomly chosen interictal period, i.e. subtract the mean and divide by the maximum of an interictal period such that a signal (interictal or octal) 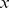 becomes 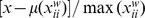, where 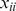 is an interictal signal and 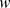 a predefined time window within it. An example of the normalised signal and dynamic/static thresholds is show in Fig. 8.

### Optimisation and Training Process

**Figure 9:**
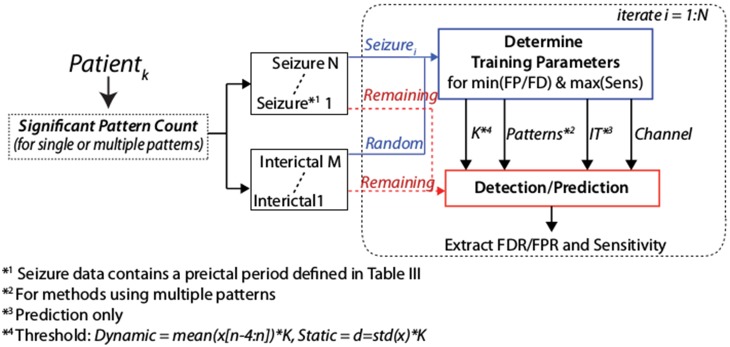
The optimisation process whereby for a single patient one seizure and one randomly selected interictal period are used for training the threshold (parameter 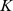), the patterns to use (in a multi-pattern method), channel and Intervention Time (prediction only). This is iterated for each seizure of the patient.

The detection or prediction threshold defined in the previous section among other parameters is determined in a training phase (Fig. 9). To determine a statistical spread of the prediction/detection accuracy we iteratively use each available seizure as the training seizure in this process. The seizure and an interictal period (typically one hour) are used to determine a threshold that minimises false predictions/detections and maximises sensitivity for that seizure and chooses the optimal data channel (see *Data* section). Once a threshold is determined it is applied to all remaining seizures and interictal periods for quantification of the prediction and detection results.

Since some of our methods will produce multiple pattern counts (Table 1), in the process of training an optimal threshold the number of pattern sizes to be used is determined. Subsequently, a detection or prediction is only counted when it occurs in a certain percentage of the selected patterns. The number of detections (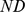) within each pattern must be above a percentage of number of patterns (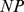) selected, given as 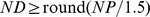. Therefore if 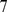 patterns are chosen then a prediction is required in 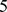 out of the 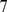 patterns to be counted.

Other parameters to be optimised include the intervention time (IT) and seizure occurrence period (SOP), both of which are defined in the next section.

### Statistical Analysis

For detection and prediction we use the standard metrics of sensitivity and false detection/prediction rate (FDR/FPR) to quantify accuracy. In addition, we use the prediction statistical framework described in [40] where a SOP and IT (or seizure prediction horizon) is defined before quantification of results. The SOP is defined as the period of time in which a seizure can occur, and the IT is the period of time before the SOP window.

To validate the results of our prediction we apply the statistical framework described in [41] and recently used in [16], [17]. Specifically this is the use of a binomial probability distribution defined by the maximum FPR and SOP and number of features (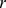) and electrodes used (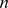) to define the probability of predicting 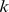 of 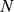 seizures.
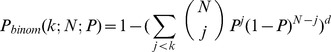
(3)where 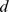 is the independent features given as 
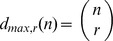
 and 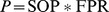 [40], [41]. Note this assumes that seizures are independent events. Given the length of interictal periods we are assuming this to be the case. The upper and lower sensitivity of a random predictor are determined by 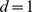 and 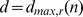 respectively with 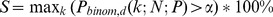 where 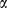 is a significance level of 0.05. More details can be found in [41]. This method allows us to quantify the significance of our prediction results.

### Data

Seizure data were obtained from the University Hospital of Freiburg Epilepsy Centre, Germany (see *acknowledgements*). The data (described in [40]) is pre-sampled at 256 Hz and quantized using a 128 channel 16-bit data acquisition. Each patient had neocortical (grid and strip electrodes) or depth electrodes and exhibited simple partial, complex partial and general tonic clonic seizures located mainly in the frontal and temporal lobe and recorded from 3 focal electrodes. This work is based on analysis of all 21 patients from this data set, encompassing a total of 87 seizures and 623 hours, a breakdown of which is shown in Table 2. The files for these data sets are separated into approximately hour segments. As not all data has contiguous segments we extract all (depending upon what is available) of the available preseizure data.

**Table 2:** Summary of patient data used in this study, including number of seizures, seizure origin, electrode type and interictal hours used.

Patient	Seizures	Precital/hrs	Origin^1^	Electrode^2^	Interictal/hrs
1	4	4.65	F	g,s	23.00
2	3	3.62	T	d	24.00
3	5	6.65	F	g,s	24.00
4	5	5.51	T	d,g,s	24.00
5	5	6.03	F	g,s	24.00
6	3	3.31	T/O	d,g,s	24.00
7	3	4.08	T	d	24.61
8	2	2.68	F	g,s	23.16
9	5	6.10	T/O	g,s	23.93
10	5	7.05	T	d	24.46
11	4	4.65	P	g,s	24.05
12	4	5.91	T	d,g,s	24.81
13	2	2.38	T/O	d,s	24.00
14	4	5.41	F/T	d,s	23.86
15	4	6.13	T	d,s	24.00
16	5	6.53	T	d,s	24.00
17	5	8.80	T	s	24.07
18	5	8.20	F	s	22.87
19	4	4.60	F	s	24.38
20	5	8.42	T/P	d,g,s	25.62
21	5	7.66	T	g,s	23.94

1 Origin  = {F: Frontal, T: Temporal, O: Occipital, P: Parietal}.

2 Electrode  = {g: grid, s: strip, d: depth}.

### Implementation

**Figure 10:**
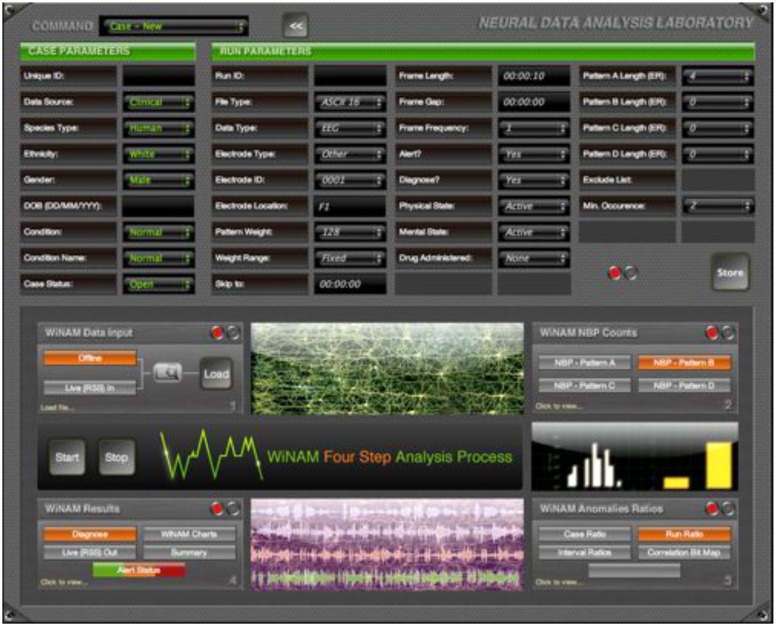
The freely accessible graphical interface designed to analyse further data sets - from www.winam.net **.**

The implementation of all methods described was carried out in Matlab v.7.14. An online analysis tool (www.winam.net) based on this code has recently been implemented (GUI shown in Fig. 10). The structure is such that the data sourcing can be through an RSS feed, or offline data sources. In both implementations the processing (N-gram) is implemented through a separate processing cluster allowing multiple parallel processing efficiently. The system is designed to vary all parameters efficiently including relevant thresholds, pattern lengths, time intervals, quantization weights etc. This is in combination with a database to allow users to input metadata.

In summary, the method we employ involves thresholding of extracted pattern counts within the data. For prediction, once a pre-seizure marker is detected, the pre-defined IT and SOP allow us to evaluate if the marker was indeed accurate in predicting the seizure. Detection does not use these pre-defined windows and as such it is simply a yes/no decision as to whether the threshold crossing is at the time of a seizure. Detection and prediction were tested as two separate studies, where optimisations were to maximise detection and prediction accuracy independently. Future work will look to combine the two methods such as to obtain an optimal threshold for both.

## Results

The results are based on pattern sizes of 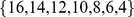 and analysis of independent channels and combinations of them with a window size of 60 seconds. This was found to be optimal to capture the seizures of which the average duration was 1.8 minutes (range 0.5 to 2 minutes). We also note that in many of the plots we indicate the results for the first seizure used in the training/optimisation process and the training seizure that produced the best results.

### Quantisation

**Figure 11:**
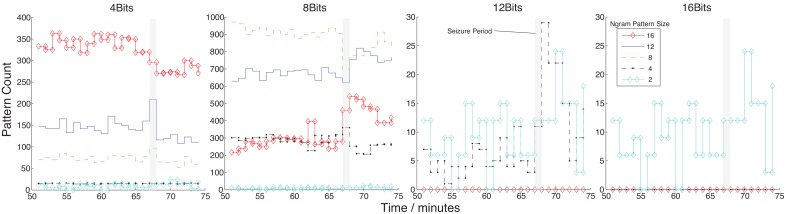
The pattern counts generated for Ngrams of 2 to 16 for various re-quantizations from the original 16 bit to 4, 8 and 12. This is done over an example seizure period to identify the most effective quantization resolution.

Prior to our selection of parameters we performed empirical studies that varied the re-quantization parameter and looked at the pattern counts generated with a variety of pattern sizes (Fig. 11). As can be seen, as we increase the resolution of our signal, we do not generate any meaningful patterns. While as low as 4 bits we see changes in many pattern counts that reflect the seizure change. Even at higher resolution (e.g. 16 bits) the patterns generated do not appear meaningful in relation to seizure onset.

There are many considerations we can explore with regards to the use of quantization as a symbolic representation. As discussed in [32], in biological data, noise is considered to be a consequence of many sources and then hence highly dimensional, as opposed to seizures that can be considered as a result of localized sources. It is this assumption amongst a few others that allows us to use methods such as phase-space reconstruction and embedding.

In the detection of activity related to seizure onset, in part this assumption holds true; the seizure is a result of various levels of synchronous activity. In prediction, as we do not know the source of activity, whether it is embedded within the noise or is a true low-dimensionality source it is difficult for us to explicitly state the implications of quantization.

Our interpretation of the results is that for synchronous spiking activity the effect of re-quantization is to capture the periodicity and shape of these spikes. The addition of the hamming distance (or NW algorithm) also improves on this activity capture. Other types of activity (e.g. high frequency, low amplitude) seen during seizures also reflect this paradigm of thought. It is our belief, of why this method generally works for detection. For prediction, it is difficult to say as we do not know all the mechanisms that lead to seizure onset. As 8 bits (i.e. 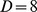) yielded the most variation in meaningful patterns we chose this for the following results.

### Method Comparison

We described *four* methods of pattern extraction for detection and prediction. We first determine which method is generally the best performer so we can then analyse the results in more details. The data consists of 21 patients, each with 2–5 seizures. Of the 6 intracortical electrodes we only analysed the 3 focal channels (in the vicinity of seizure onset). We assess the detection and prediction sensitivity and FDR/FPR averaged for each patient over all channels and over all combination of training and test seizure.

**Figure 12:**
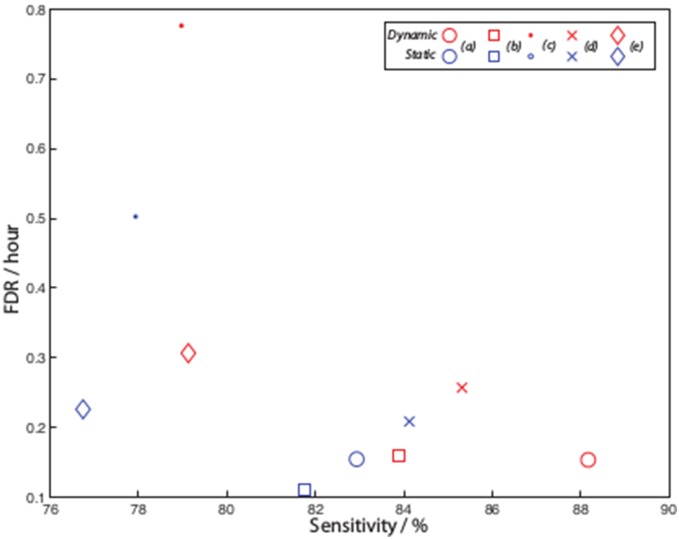
Comparison of detection sensitivity versus FDR for several variations on pattern methods (Section 0), including (a) multiple patterns (16, 14, 12, 10, 8 and 6, method *(3)*), (b) multiple patterns with overlaps (method *(4)*), (c) overlapping patterns of size 12 (method *(2)*, and two non-overlapping patterns (method *(1)*), (4) 12 and (5) 6. Results for the static and dynamic (moving average) threshold are also shown.

**Figure 13:**
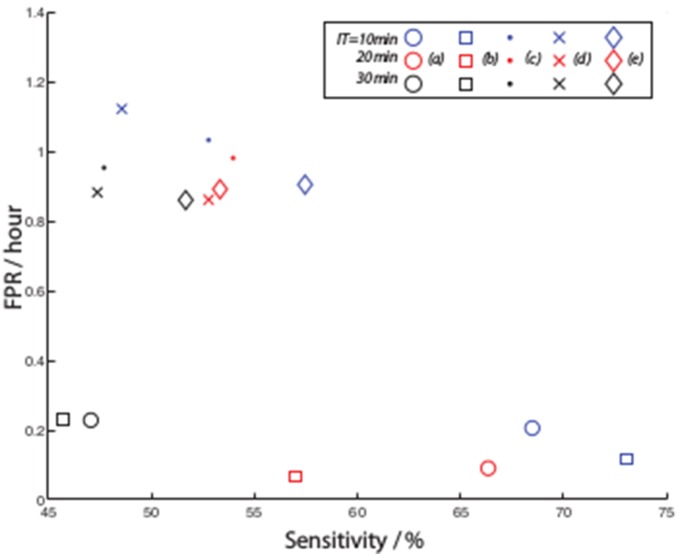
Comparison of prediction sensitivity versus FPR for the same variations on pattern methods depicted in Fig. 12.

The detection and prediction results show that the methods using multiple patterns (*(3)* and *(4)*) perform best in sensitivity and FPR/FDR (Figs. 12 and 13). The others still achieve some relatively high sensitivities but with poor FDR and FPR. As in general method *(4)* outperforms the others the remainder of the results will be focused on this. Similarly a dynamic threshold is found to outperform a static one in detection, and although not shown, in prediction.

### Detection

**Figure 14:**
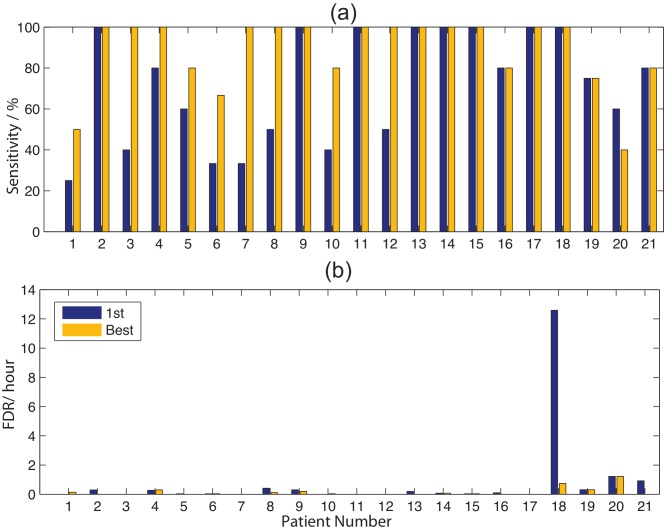
For each channel, the (a) sensitivity and (b) FDR are shown for the minimum FDR result for the best case and 1st seizure.

Both the first seizure used for training and the best performing training seizure for the optimal channel are shown in Fig. 14.

These results show us that: (1) Assuming we only use the first seizure, we would have a sensitivity of 71.8% with FDR of 0.8. This is primarily distorted by case 18, that without sensitivity becomes 70.33% and FDR of 0.212. Assuming we have multiple seizures to fine tune the process we can achieve a sensitivity of 88.17% with FDR of 0.15.

We note that any combination of FDR and sensitivity can be implemented. For example, case 18, a better result uses the second seizure for optimization, giving a sensitivity of 20% and an FDR of 0. In addition, as we do not have information about the specific seizure types, or whether they are clinical or subclinical thus cannot illustrate in more detail why some seizures are detected and others not. The lack of accurate databasing with well annotated data sets has prompted initiatives to create them, including the Epilepsiae project [42], [43].

### Prediction

**Figure 15:**
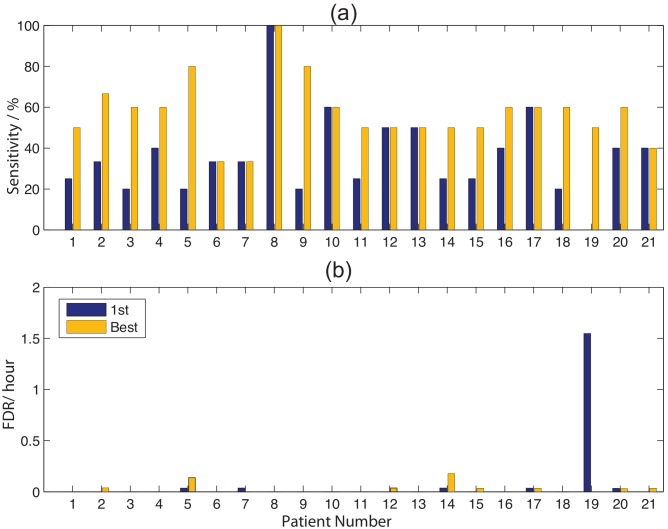
The (a) sensitivity and (b) False prediction rate for the optimal channel with the best and 1st seizure used for training, optimised across all ITs.

For prediction, the same parameters were used and, given the limited preseizure time available (due to the non contiguousness of the data samples), we analysed intervention times (IT) of 30, 20 and 10 minutes and with an SOP of 10 minutes. An example of the results for the 20 minute (SOP = 10 minutes) period is shown in Fig. 15, and for patient 15, the pattern change for a pattern size of 6 is shown in Fig. 8.

Tables 3 and 4 summarise the sensitivitities using the best and 1st seizure training case for each of the ITs and combining them to minimise FPR and maximise sensitivity. As expected, maximising sensitivity increases FPR and interestingly shorter ITs result in higher sensitivity results.

**Table 3:** Sensitivity and FPR for an IT of 30, 20 and 10 minutes and for the best and 1st seizure training case for an SOP of 10 minutes.

SOP = 10 mins										
IT (mins)	10		20		30		Opt. (Min. FPR)		Opt. (Max. Sens)	
	*S* 1	*FPR* 2	*S*	*FPR*	*S*	*FPR*	*S*	*FPR*	*S*	*FPR*
**Best case**	68.49%	0.21	66.35%	0.09	47.06%	0.23	57.30%	0.03	75.16%	0.21
**1st case**	46.51%	0.31	50.48%	0.28	30.71%	0.28	36.19%	0.10	59.44%	0.28

Also displayed are the optimal statistics when minimising FPR and maximizing sensitivity.

1 S: Sensitivity.

2 FPR: False Prediction Rate.

**Table 4:** As in Table 3 showing sensitivity and FPR for an IT of 30, 20 and 10 minutes and for the best and 1st seizure training case for an SOP of 20 minutes.

IT (mins)	10		20		30		Opt. (Min. FPR)		Opt. (Max. Sens)	
	*S^1^*	*FPR^2^*	*S*	*FPR*	*S*	*FPR*	*S*	*FPR*	*S*	*FPR*
**Best case**	90.95%	0.06	70.87%	0.06	58.57%	0.11	78.33%	0.01	93.81%	0.06
**1st case**	70.08%	0.21	50.08%	0.12	37.78%	0.11	54.37%	0.09	72.22%	0.22

To differentiate these results from a random predictor we use the binomial probability statistic with an FPR of 0.15 and SOP of 10 minutes to define our critical sensitivity boundaries (Section *Statistical Analysis*). We break down the results similar to that presented in [17] where the results are separated by the seizure focal region. This shows us that the highest FPR are contributed by the frontal region patients and a large proportion (greater than 80%) show significance (Table 5). Interestingly, this is for the 1st case seizure, but for the best case the results show that, for an SOP of 20 mins and maximising sensitivity, we can achieve 100%, 76.4% and 95.8% sensitivities and FPRs of 0.05, 0.05 and 0.10 for temporal, frontal and the remaining (others) respectively, all exceeding a random predictor.

**Table 5:** The sensitivity, FPR and number of patients that exceed the lower and upper critical sensitivity (and FPR less than 0.15) for different brain focal onset regions for an SOP of 10 and 20 minutes.

SOP = 10 min	1ST: min. FPR				1ST: max. S			
	Statistics		Exceeds		Statistics		Exceeds	
**Temporal**	42.41%	0.01	77.8%	55.6%	66.85%	0.12	100%	88.9%
**Frontal**	30.83%	0.31	83.33%	83.33%	71.67%	0.61	100%	100%
**Other**	32.22%	0.01	83.33%	33.33%	51.94%	0.09	100%	50.00%
**SOP = 20 min**								
**Temporal**	52.78%	0.00	88.89%	55.6%	67.41%	0.04	100%	88.89%
**Frontal**	55.00%	0.30	83.33%	83.33%	71.67%	0.61	100%	100%
**Other**	56.11%	0.01	100%	100%	80.00%	0.08	100%	100%

### Comparison to Previous Studies

A select few studies have utilised the same data sets for the same goal, seizure detection and prediction. This section reviews the results of these to offer a reliable comparison with our own results. Recently Zhou et al [44] showed a sensitivity of 96.25% and a FDR 0.13/h when using linear discriminant analysis and lacunarity for seizure detection, one of the highest results from those mentioned earlier.

The data set has mainly been used, for seizure prediction, by groups at Freiburg. The first two, from 2003, analysed the dynamic similarity index (DSI) [40] and phase synchronisation [2]. The *dynamic similarity index*, was able to achieve, for a maximum FPR of 0.15 and SOP of 30 minutes, sensitivities of around 40%. The maximum sensitivity for this FPR was around 50% at an SOP of 36 minutes. The second [2], used phase synchronisation to achieve prediction sensitivities of 8.3–38.3% with an FPR of 0.1/h. As expected larger FPRs result in larger sensitivities; FPR of 0.6/hour and 90% sensitivity. An interesting result of this work was that, firstly, smaller prediction windows performed worst and that hippocampal showed much greater sensitivity that cortical; hippocampal average at around 95% sensitivity at an FPR of 1/hour, while neocortical was slightly above 80% for the same FPR.

In 2004, a comparison was performed between the DSI, correlation dimension and accumulated energy [45]. They showed that the DSI was the best of all methods, showing an FPR of 1–3.6/day (0.04–0.15/hour) and sensitivity of 21–42%. All results were considered significant against a random predictor.

More recently, in 2006, two studies considered further analysis of this data set using the DSI [41] and mean phase coherence [46]. The former, focused on the first 4 patients’ data and showed sensitivities as a function of IT, SOP and FPRmax. For an SOP of 30 minutes and IT of 10 minutes, they achieve a sensitivity of 40%, with a FPR of 3/day (0.125/hour). They show that a sensitivity of 100% can be achieved for an FPR of 1/hour, although only slightly greater than the upper critical sensitivity of a random predictor. It is interesting to not the large patient variability (sensitivities of 40–80%). Finally, the phase coherence and DSI were tested and showed high sensitivities (around 80%) for an FPRmax of 0.5/hour. Interestingly they showed that most false predictions occurred at night. DSI was found to perform the best and an SOP of 30 minutes and IT of 2 minutes.

These results, comparable to our own, do show some interesting outcomes, especially those related to variation in results depending on brain region and time of day, as well as inter-patient variability and the optimisation of FPR, SOP, IT and sensitivity.

## Discussion

The method we have applied here extracts and counts the number of repeating patterns in a fixed time window. Hence a pseudo-periodic feature, linear or nonlinear, could be counted. The nonstationary nature of EEG make the alignment and extraction of these patterns much more difficult, but is alleviated by the utilisation of similarity quantifiers (Hamming distance and NW algorithm). Using intracranial EEG allowed for an overall better signal-to-noise ratio than that of scalp recordings and although the data did still contain artefacts these were eliminated by Savitsky-Golay filtering.

Several improvements can be made to the method to alleviate some potential drawbacks. These include: (1) between windows no attempt has been made to assess whether particular patterns are consistent between them and how specific patterns may be more important detective/predictive markers over others, (2) using overlapping windows needs to be investigated to see whether this avoids any discontinuities in the pattern counts, (3) finally, to analyse larger, annotated data sets such that we can correlate patterns to different types of seizures. We also aim to use these data sets for training across patient sets, i.e. using patient seizure data to predict/detect other patients and seizures. It is interesting to note that false detections and predictions are higher in frontal cases (patients 18 and 19). This could be due to increased artefacts and general activity typically found in this area of the brain. Better classification of artefacts will be required to better quantify the results as well as considerations from previous studies, on time of day and cortical versus hippocampal seizures.

Although this is a computational method, the actual processing is fixed point and therefore lends itself to implementation in traditional Von-Neumann architectures and parallel processing, making it extremely efficient for reconfigurable (e.g. FPGA) and custom (e.g. ASIC) hardware implementations. Accuracy vs. complexity of the algorithm needs to be explored, including utilisation of the electrographic to clinical onset time for more accurate quantification of detection. Given also that better thresholds can be defined based on the use of multiple seizures, our methodology would be suitable for self-learning systems that optimise based on previous events and activity.

Further to this, the results show that the seizure that produce the highest sensitivity and FPR is not typically the first. This implies that our method is best suited to longer training phases using multiple seizures to train parameters. Hence our desire to use more extensive (and continuous) data sets with multiple seizures and training phases. Although training was employed the parameters, including the threshold, can be better defined. It may be better suited to define a threshold based on the several hours of interictal data and then apply this to ictal periods, i.e. defining what is normal so you can identify abnormalities.

## Conclusions

We have presented a new approach that utilises elements of N-grams and symbolic signal representation schemes combined with sequence similarity metrics to track dynamical changes in the various ictal states. Using intracranial EEG recordings we were able to quantify the detection and predictive power of this method using simple thresholding schemes. We assessed our method using standard statistical measures of sensitivity and false prediction rate using a single seizure and one hour interictal period to train this threshold and the optimal pattern lengths to use for a specific patient. The non-contingent nature of the data led us to use the binomial probability critical sensitivity tests [16], [17] over surrogate data analysis as the method for quantifying statistical significance of our results.

This work has successfully demonstrated an N-gram based algorithm with significant predictive power. With an average sensitivity of 67% for temporal lobe seizures and FPR of 0.04 for an SOP of 20 minutes and combined ITs of 30, 20 and 10 minutes. Frontal seizures brought the increased the average FPR, showing 72% sensitivity and FPR of 0.61 when maximizing sensitivity. This led to an overall maximum average of 75.16% and 0.21 FPR. Using different seizures for training yielded much higher results, warranting the use of multiple training seizures for future work. For temporal cases this means a sensitivity of 100% is achievable and on average low false predictions (0.06, with almost all cases exceeding the upper critical sensitivity). This showing that this method of prediction has significant predictive power that warrants further study.
